# Resource-Saving Customizable Pipeline Network Architecture for Multi-Signal Processing in Edge Devices

**DOI:** 10.3390/s22155720

**Published:** 2022-07-30

**Authors:** Ping Song, Youtian Qie, Chuangbo Hao, Yifan Li, Yue Zhao, Yi Hao, Hongbo Liu, Yishen Qi

**Affiliations:** 1 The Key Laboratory of Biomimetic Robots and Systems, Ministry of Education, Beijing Institute of Technology, Beijing 100081, China; sping2002@bit.edu.cn (P.S.); qieyoutian@bit.edu.cn (Y.Q.); yifanli@bit.edu.cn (Y.L.); 3220210562@bit.edu.cn (Y.Z.); 3120190156@bit.edu.cn (Y.H.); 3220200138@bit.edu.cn (H.L.); 1120182770@bit.edu.cn (Y.Q.); 2 The Beijing Jinghang Computation and Communication Research Institute, Beijing 100074, China

**Keywords:** edge computing, signal processing, pipeline network architecture, FPGA

## Abstract

With the development of the information age, the importance of edge computing has been highlighted in industrial site monitoring, health management, and fault diagnosis. Among them, the processing and computing of signals in edge scenarios is the cornerstone of realizing these scenarios. While the performance of edge devices has been dramatically improved, the demand for signal processing in the edge side has also ushered in explosive growth. However, the deployment of traditional serial or parallel signal processing architectures on edge devices has problems such as poor flexibility, low efficiency, and low resource utilization, making edge devices unable to exert their maximum performance. Therefore, this paper proposes a resource-saving customizable pipeline network architecture with a space-optimized resource allocation method and a coordinate addressing method for irregular topology. This architecture significantly improves the flexibility of multi-signal processing in edge devices, improves resource utilization, and further increases the performance potential of edge devices. Finally, we designed a comparative experiment to prove that the resource-saving and customizable pipeline network architecture can significantly reduce resource consumption under the premise of meeting real-time processing requirements.

## 1. Introduction

Edge computing refers to the provision of computing processing services on the side close to the source of objects or data [1,2]. Thanks to the low power consumption, small size, and high performance of edge devices, edge computing is being widely used in various fields, such as signal denoising [3], frequency domain analysis [4], industrial field condition monitoring [5,6], fault diagnosis [7,8], health management [9,10], feature extraction [11], signal solving [12], etc. Among them, the processing and computing of signals in edge scenes is the key to realize the functions of these scenes. According to the results of signal processing and computing, the edge device returns the result to the control terminal or performs corresponding actions to realize edge intelligence. With the rapid growth of the demand for edge computing, the number and types of signals to be processed have further increased, and the complexity of signal processing has further increased. Therefore, the architecture of signal processing has ushered in new challenges.

Generally speaking, low-speed signal processing uses single-chip microcomputers [13], while high-speed signal processing generally uses ARM processors [14]. When there are a lot of high-speed signals that need to be collected and processed, it is difficult for single-chip computers or ARM processors to meet the needs. Especially with the rapid development of fault diagnosis and health management in recent years, the number of signals that need to be collected and processed in a single system is also rapidly increasing. The traditional serial signal processing architecture is no longer competent.

**Figure 1:**
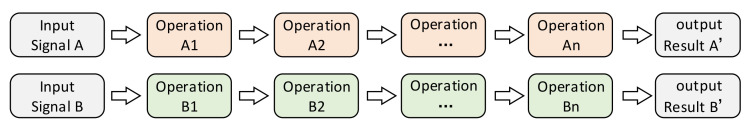
Traditional multi-signal parallel processing method.

With the rapid development and application of the field programmable gate array (FPGA) in recent years, it has been widely used in high-speed parallel acquisition and high-speed parallel processing [15,16,17]. However, this method also has some problems. In different scenarios, the signals that need to be processed are different, and the desired results are different. Signal processing needs to be redesigned [18]. At the same time, most of the multi-channel signal processing designed by FPGA are simple channel superpositions, as shown in Figure 1, which wastes a lot of resources [19,20]. Therefore, this method is not suitable for scenarios with limited edge resources.

**Figure 2:**
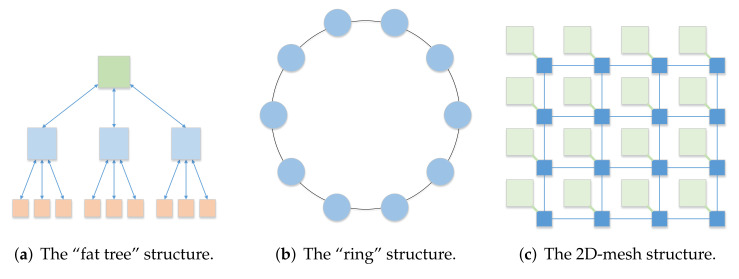
Different types of NoC structures.

Each signal processing method generally has multiple different processing flows, and the same or different signal processing methods may have the same processing unit. A network-on-chip (NoC) is used to connect different resources in the form of a network. Both have certain similarities. We regard each operation of signal processing as a resource, and use the idea of a NoC to connect these resources. Therefore, to realize the reuse of resources and improve the utilization rate of resources, we have studied several common structures of NoC systems. Traditional single-chip processors use a bus-type on-chip network structure. When an operation occupies bus resources, other operations cannot be performed. This structure performs poorly in parallelism. The “fat tree” structure [21] adopts a tree-like structure. The computing unit is located at the leaf position of the tree, and the branches of the tree are used for addressing the problem, as shown in Figure 2a. The structure is simple, but the flexibility is poor, and thus, it is not suitable for large-scale parallel processing scenarios. The “ring” and the improved structure [22] adopt a ring structure, as shown in Figure 2b. When the number of ring nodes increases, the network diameter increases, and the communication delay increases. This structure is prone to blocking parallel signal processing. The 2D-mesh structure [21] is one of the most widely used NoC structures, as shown in Figure 2c, which adopts a two-dimensional array. It is more flexible and has better parallelism. However, the routing design is more complex, and routing takes up more resources. The Torus structure [23] adds ring routing to 2D-mesh, which further improves routing flexibility and consumes more resources for routing.

In summary, the multi-signal processing requirements of edge devices are complex and require high real-time performance. Moreover, edge devices are limited in performance and power consumption. The traditional serial architecture has problems such as low execution efficiency and long multi-signal processing time. The traditional parallel architecture has problems such as more resource occupation and poor flexibility. The idea of NoC provides a new idea for the multi-signal processing of edge devices. However, the current research on the NoC structure is mainly aimed at available on-chip multi-core systems. The uncertainty of the routing path is high, the routing structure is complex, and it occupies many hardware resources, so it is not suitable for multi-signal processing of edge devices. In this paper, based on the above research, we propose a resource-saving customizable pipeline network architecture for multi-signal processing in edge devices. The main contributions of this paper are summarized as follows:

1. This paper proposes a resource-saving customizable pipeline network (RSCPN) architecture. This architecture significantly improves the flexibility of multi-signal processing in edge devices, improves resource utilization, and further increases the performance potential of edge devices.
2. This paper proposes a space-optimized resource allocation method for RSCPN. Under the premise of comprehensively considering the reusability of processing units, execution time of different processing units, resource occupancy, real-time performance and other factors, the method realizes the optimal allocation of space resources.
3. This paper designs a flexible and customizable pipeline routing unit, establishes a resource-saving irregular topology, and proposes a coordinate addressing method for irregular topology. The method reduces the useless paths of the traditional routing topology and reduces the consumption of routing resources to ensure the flexibility of signal pipeline processing.

The remainder of this paper is organized as follows. Section 1 introduces the current status of the signal processing method and network-on-chip technology and proposes the research content of this article. Section 2 introduces the resource-saving customizable pipeline network architecture. Section 3 introduces the comparative experiments of RSCPN and other methods. Section 4 analyzes the advantages and disadvantages of different methods based on the experimental results, and Section 5 summarizes the paper’s conclusion.

## 2. Resource-Saving Customizable Pipeline Network Architecture

**Figure 3:**
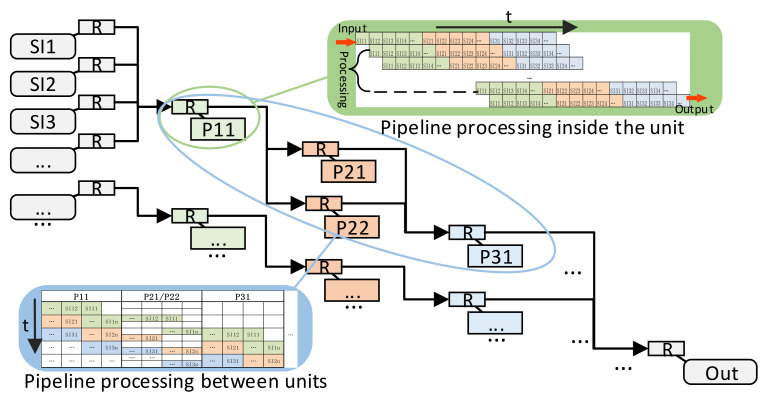
The resource-saving customizable pipeline network architecture.

In view of the fact that the multi-signal processing process is relatively fixed, and different signals may require the same processing unit, combined with the idea of the network-on-chip, we have designed a resource-saving customizable pipeline network architecture for multi-signal processing in edge devices, as shown in Figure 3.

This structure regards the signal processing unit as a resource and uses a customizable pipeline routing between resources to realize data transmission, which significantly improves the flexibility of pipeline signal processing. At the same time, the structure uses the pipeline routing unit to multiplex the processing units with the same function, which reduces resource consumption. Each processing unit adopts a pipeline structure, which can support the continuous processing of pipeline data, as shown in the green part of Figure 3. The data transmission between the routing units also adopts the pipeline structure. The routing unit does not cache data, but only caches the routing information of the data so that the data can be processed and output at the same time, as shown in the blue part of Figure 3. The traditional NoC routing unit generally needs to wait for data packet transmission to be completed before processing, and its transmission time will significantly increase the overall processing time. In this structure, the transmission and processing of data are carried out simultaneously in the form of pipelines, and the transmission time has little influence on the overall processing time.

### 2.1. Space-Optimized Resource Allocation Method

In order to reduce the resource occupancy of RSCPN for multi-signal processing, we propose a spatially optimal resource allocation method. Suppose a processing system has *N* input signals, $S=\{s_{1},s_{2}\ldots ,s_{n}\}$, and the corresponding sampling rate $Sap=\{sap_{1},sap_{2},\ldots ,sap_{n}\}$. Under normal circumstances, the data sampling speed will be lower than the data processing speed, so we generally package the data first and then process it to reduce the waiting time consumption and improve the utilization rate of hardware resources. The amount of data to be processed in each signal is $PKGn=\{pkgn_{1},pkgn_{2},\ldots ,pkgn_{n}\}$. We obtain the ready time of each packet $Tpkg=\{tpkg_{1},tpkg_{2},\ldots ,tpkg_{n}\}$ according to the sampling rate:

(1) $$\begin{array}{c} Tpkg=\frac{PKGn}{Sap} \end{array}$$

where $t_{c}$ represents the period in which all signals will appear an integer number of times and at least once. $t_{c}$ should be equal to the least common multiple of TPKG:

(2) $$\begin{array}{c} t_{c}=LCM\left(tpkg_{1},tpkg_{2},\ldots ,tpkg_{n}\right) \end{array}$$

Assume that there are *m* different processing units PU in the above signal processing.

(3) $$\begin{array}{c} PU=\{pu_{1},pu_{2},\ldots ,pu_{m}\} \end{array}$$

A processing unit may be used in multiple signal processing. $PUs_{i}$ represents the processing units $PU_{i}$ used in *k* signals:

(4) $$\begin{array}{c} PUs_{i}=\left\{s_{ui1},\ldots ,s_{uik}\right\},k\in \left[1,n\right] \end{array}$$

The time occupied by each operation $TPU_{i}$ is positively related to the complexity of the operation $O(PU_{i})$ and the length of the data processed by the operation. We can use this feature to estimate the maximum time for each operation.

(5) $$\begin{array}{c} TPUm_{i}\propto \left\{tpkg_{i},O\left(PU_{i}\right)\right\},i\in \left[1,n\right] \end{array}$$

According to Equations (4) and (5), the total processing time required by the processing unit in a certain cycle can be calculated as

(6) $$\begin{array}{c} TPUp_{i}=\sum_{j=1}^{k}TPUm_{uij}\times \frac{t_{c}}{tpkg_{uij}} \end{array}$$

In order to avoid the problem that a processing unit is blocked due to too many tasks, the following conditions must be met:

(7) $$\begin{array}{c} \forall TPUp_{i}\leq t_{c},i\in \left[1,n\right] \end{array}$$

To increase the robustness of resource planning, we add a redundancy factor θ to the above equation. The general value range of θ is (0.8, 1).

(8) $$\begin{array}{c} \forall TPUp_{i}\leq t_{c}\times \theta ,i\in \left[1,n\right] \end{array}$$

If there is $TPUp_{i}$ that does not satisfy Equation (8), we increase the number of $PU_{i}$ in this system:

(9) $$\begin{array}{c} N_{pu_{i}}=\left\lceil \frac{t_{c}}{TPUp_{i}}\right\rceil \end{array}$$

We evenly distribute the original signal to be processed by $PUs_{i}$ in Equation (4) to $N_{pu_{i}}$$PUs_{i}$. We then loop through all $PUs_{i}$ until all $PUs_{i}$ meet the above conditions. Then, these processing units are connected by pipeline units in processing order.

In addition, when some processing units have simple logic and occupy fewer resources, it may happen that the resources saved are less than the resources consumed by the pipeline routing unit:

(10) $$\begin{array}{c} Rpu_{i}\times N_{pu_{i}}<Rpr_{i} \end{array}$$

where $Rpu_{i}$ represents the resources occupied by the processing unit *i*, and $Rpr_{i}$ represents the resources occupied by the corresponding routing unit *i*. In this case, we can implement the corresponding signal processing by direct connection without using the pipeline routing unit.

### 2.2. Pipeline Structure Establishment and Coordinate Assignment of Processing Units

**Figure 4:**
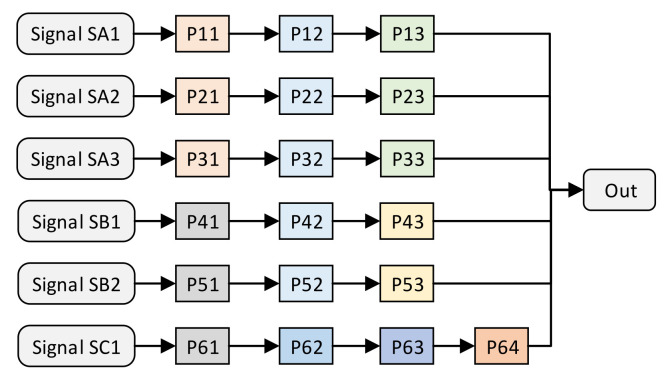
An assumption of the independent multi-signal processing method.

The multi-signal processing unit forms an irregular pipeline network structure after the space-optimized resource allocation method. We use an example to describe the pipeline structure establishment and the coordinate assignment of the processing units. Suppose that there is such a requirement for multi-signal processing independent of each other, as shown in Figure 4. Each signal undergoes multiple signal processing operations to obtain the output result; for example, sa1 needs to go through P11, P12, and P13 to obtain the output result. The ID of the processing unit is marked by 2D coordinates. The color of the processing unit in the figure represents the type of processing unit. Processing units of the same color have the same function; for example, processing units P11, P21, P31 have the same function.

**Figure 5:**
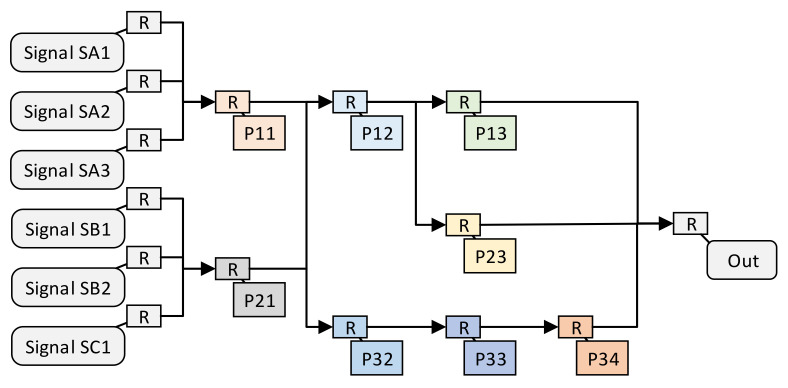
The pipeline structure of the independent multi-signal processing method.

Assuming that these same processing units meet the constraints of the spatially optimal resource allocation method, the structure of the independent multi-signal processing method after the spatially optimal resource allocation method is shown in Figure 5. In order to improve the utilization of coordinate IDs, we rearranged the IDs in Figure 4. First of all, we divided the signals with the same processing operation into a group; then, the 6 signals can be divided into 3 groups, G1,G2, and G3, as shown in Equation (11).

(11) $$\begin{array}{cc} G1 & =\{SA1,SA2,SA3\}\\ G2 & =\{SB1,SB2\}\\ G3 & =\{SC1\} \end{array}$$

Then, the processing units of each group are numbered according to the 2D coordinates. The abscissa represents the group num($G_{x}$); for example, the abscissas of the processing units in G1, G2, and G3 are [1,2,3], and the ordinate represents the sequence of the processing units.

(12) $$\begin{array}{cc} Pg1 & =\{P11,P12,P13\} \end{array}$$

If the processing unit in the previous group is used in the current group, then the number of the previous group is used directly, as shown in Equation (13). The resulting pipeline structure of independent multi-signal processing method is shown in Figure 5.

(13) $$\begin{array}{cc} Pg2 & =\{P21,P12,P23\}\\ Pg3 & =\{P21,P32,P33,P34\} \end{array}$$

**Figure 6:**
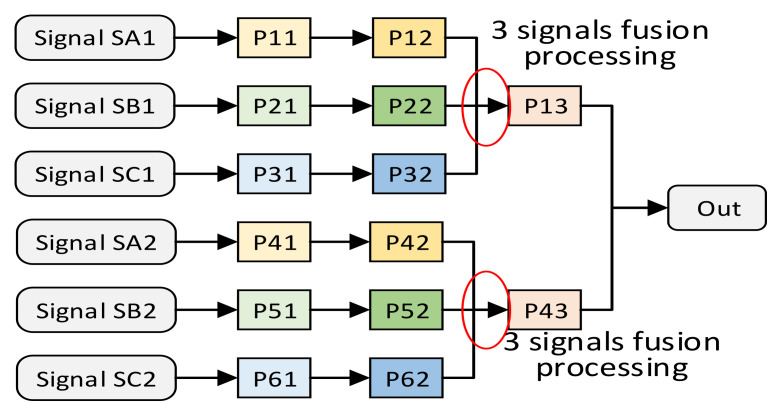
An assumption of the fusion-type multi-signal processing method.

**Figure 7:**
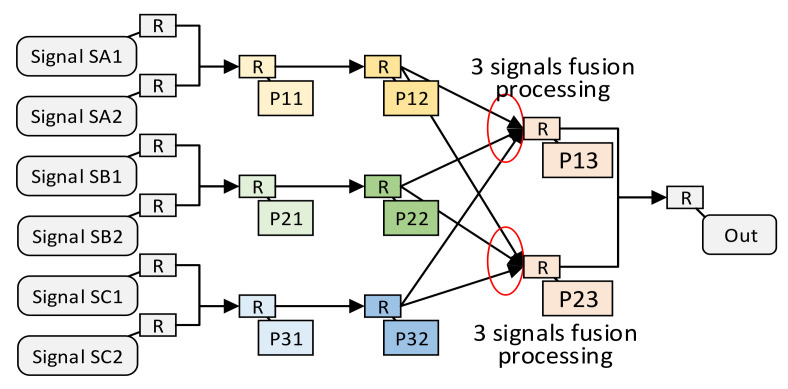
The pipeline structure of the fusion-type multi-signal processing method.

Except for the above-mentioned multi-signal independent situation, multi-signal fusion processing is also common in signal processing. We use an example of multi-signal fusion processing to show the structure of multi-signal fusion processing. Suppose there is such a requirement for multi-signal fusion processing, as shown in Figure 6. Signal SA1 first passes through processing units P11 and P12. Signal SB1 first passes through processing units P21 and P22, and signal SC1 first passes through processing units P31 and P32. Then, signal SA1, SB1, SC1 are, respectively, input into the P13 fusion processing unit to obtain the processing result. Assuming that these same processing units meet the constraints of the spatially optimal resource allocation method, the structure of the independent multi-signal processing method after the spatially optimal resource allocation method is shown in Figure 7. Due to the uncontrollable routing delay, multiple groups of signals that need to be merged and processed may not arrive at the fusion processing unit in sequence. Therefore, in order to avoid functional errors, we do not merge the fusion processing units P13 and P23.

### 2.3. Coordinate Addressing Method for Irregular Topology

Different from the traditional regular NoC structure, we cut out the unnecessary routing paths in RSCPN, which further reduces the complexity of routing addressing and the consumption of routing units. Different routing units in Figure 5 have different numbers of interfaces. The traditional coordinate addressing method cannot be used in this irregular routing structure. Therefore, on the basis of coordinate addressing, we propose a coordinate addressing method for irregular topology. Irregular routing addressing is mainly divided into two cases. In RSCPN, most routing addressing can find the corresponding processing unit at the next level of routing. To further increase flexibility, the structure also supports addressing across processing units.

#### 2.3.1. Flexible and Customizable Pipeline Routing Unit Design

**Figure 8:**
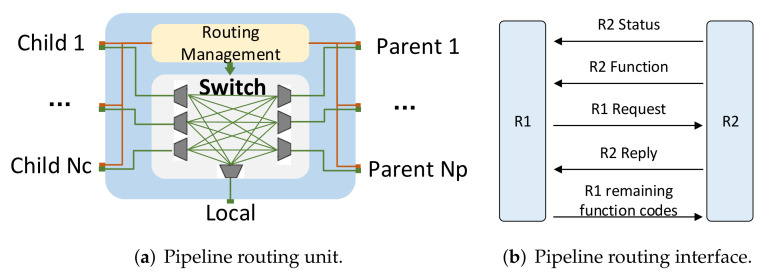
The pipeline routing unit and its interface.

Traditional NoC routing units generally have complex arbitration mechanisms, data buffers, etc., which occupy a large amount of resources. In order to meet the routing addressing requirements of irregular topologies and reduce the resource occupation of traditional NoC routing units, we have designed a resource-saving pipeline routing unit that can be flexibly tailored, as shown in Figure 8a. The unit consists of multiple groups of child interfaces ($N_{c}$), multiple groups of parent interfaces ($N_{p}$), local interfaces, switch switches, and routing management units. The structure of the child and parent interface mainly includes a data interface and routing interface. The data interface is responsible for data transmission, using the AXI bus of Xilinx; the child is the slave, and the parent is the master. The local interface is responsible for connecting with processing resources and also uses the AXI bus for data transmission. The switch is responsible for establishing the connection between child, parent and local processing resources. The routing interface is responsible for routing addressing and routing establishment, as shown in Figure 8b. “R2 Status” represents the current status of route R2; “R2 function” represents the function code of the processing unit connected to route R2 and is also the coordinates of route R2. “R1 Request” represents route R1 sending a connection request to R2; “R2 Reply” represents the result of routing R2’s reply to R1. “R1 remaining function codes” represents the remaining operations of the packet sent by routing R1.

**Figure 9:**
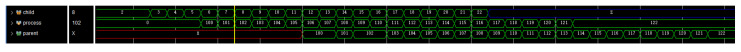
An example of simultaneous input and output. “child” represents the input data [0, 1, …, 22], “process” represents the data in the process [100, 101, …, 122], “parent” represents the output data [100, 101, …, 122].

The inside of the pipeline routing unit is also designed with the idea of the pipeline. With the pipeline processing unit, the pipeline processing unit can realize simultaneous input, processing and output operations, as shown in the Figure 9. The “child” represents the data of the input interface, and the “parent” represents the data of the output interface after processing. Different from the traditional packet routing method, the pipeline structure realizes that when data is input, it can be output at the same time, which greatly improves the efficiency of data processing.

**Figure 10:**
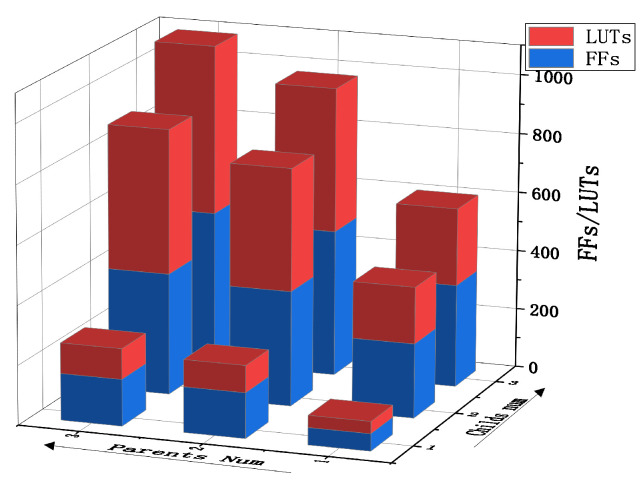
Resource occupation of pipeline routing units with different numbers of interfaces.

**Figure 11:**
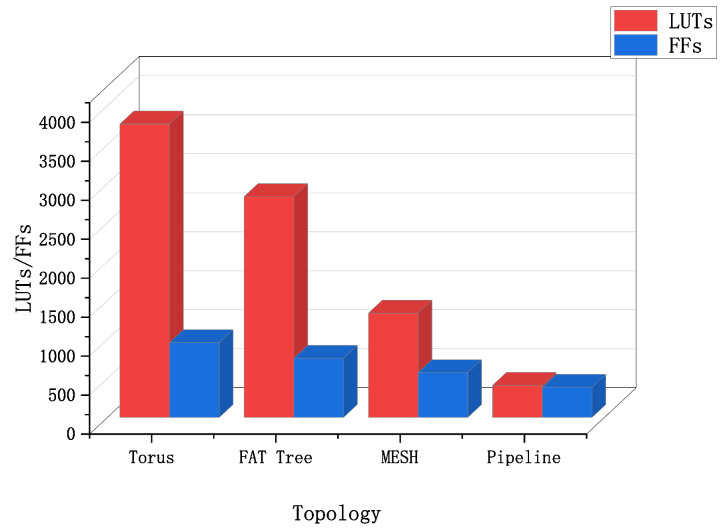
Comparison of resource occupancy of routing units with 4 interfaces in different topologies.

The pipeline routing unit can quickly obtain new routing units with different numbers of child and parent interfaces by cutting. The resource occupation of routing units with different numbers of interfaces is shown in Figure 10. Using routing units with a corresponding number of interfaces in actual use will reduce resource waste. Compared with the traditional routing unit structure of the network-on-chip, the pipeline routing unit saves a lot of resources due to its simple structure. According to reference [24], we compare the routing unit structure and pipeline routing structure of the traditional on-chip network, as shown in Figure 11. Under the same condition with 4 interfaces, the pipeline routing unit consumes the least resources. The LUTS of the pipeline routing only occupies 30.8% of the “MESH” routing. The FFS of the pipeline routing only occupies 67.2% of the “MESH” routing.

#### 2.3.2. Route Establishment Process of a Single Pipeline Routing Unit

Based on the pipeline routing unit, we design a route establishment process of pipeline processing method. The route establishment process of a single pipeline routing unit is shown in Algorithm 1.

**Algorithm 1** Route establishment process.**initial**: R(i−1): The previous routing unit;R(i): The current routing unit;R(i+1): The next routing unit;$R{\left(i\right)}_{in}=\left[R{\left(i\right)}_{in1},R{\left(i\right)}_{in2},R{\left(i\right)}_{inm}\right]$: R(i) has m input ports;$R{\left(i\right)}_{out}=\left[R{\left(i\right)}_{out1},R{\left(i\right)}_{out2},R{\left(i\right)}_{outn}\right]$: R(i) has n output ports;**input**: $R{\left(i-1\right)}_{req}$: Connection request from R(i−1);$R{\left(i-1\right)}_{rfc}$: Remaining function codes of R(i−1);$R{\left(i+1\right)}_{locf}$: R(i+1)’s local function code;$R{\left(i+1\right)}_{sts}$: R(i+1)’s current state;**output**: $R{\left(i\right)}_{rpy}$: R(i)’s reply to the request of R(i+1);$R{\left(i\right)}_{sts}$: R(i)’s current state;$R{\left(i\right)}_{locf}$: R(i)’s local function code;$R{\left(i\right)}_{rfc}$: Remaining function codes of R(i);$R{\left(i\right)}_{req}$: R(i)’s Connection request to R(i+1)
1:$R{\left(i\right)}_{sts}=0$;2:$R{\left(i\right)}_{locf}=LOCF$;  //Localfunctioncode3:for j=1:m   //Respondtoconnectionrequests4: if $R{\left(i-1\right)}_{req}$∈$R{\left(i\right)}_{inj}$≠0 && $R{\left(i\right)}_{sts}==0$5:  $R{\left(i\right)}_{rpy}=1$;  //readytotransferdata6:  $R{\left(i\right)}_{sts}=1$;7:  if$(R{\left(i-1\right)}_{rfc}\;\&\;0$x$ff==R{\left(i\right)}_{locf})$8:   $R{\left(i\right)}_{locf}{=(R\left(i-1\right)}_{rfc}>>8$;9: endif10:endfor11:if $R{\left(i\right)}_{sts}\neq 0\;\;\;\;//Send\;connection\;request\;to\;the\;next\;route\left(ignore\;local\;processing\right)$12: for k=1:n13: if$\left(\left(R{\left(i+1\right)}_{sts}\in R{\left(i\right)}_{outk}\right)==0\right)\;\&\&(\left(R{\left(i+1\right)}_{locf}\in R{\left(i\right)}_{outk}\right)==R{\left(i\right)}_{locf}\&0$xff)  14:  $R{\left(i\right)}_{req}=1$;15: endfor16:endif


#### 2.3.3. Route Establishment Process of the RSCPN

**Figure 12:**
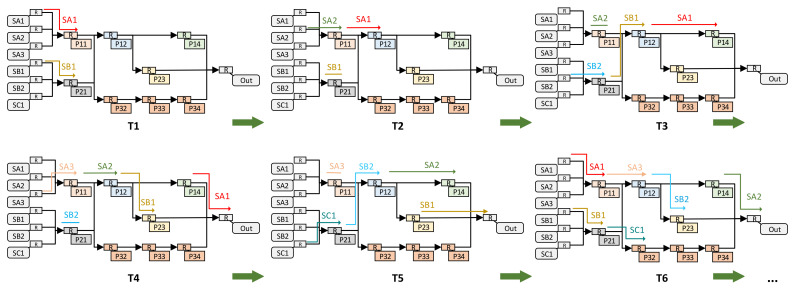
The processing flow of the pipeline method. T1–T6 in the figure represent different moments and T1 < T2 < T3 < T4 < T5 < T6.

We use the structure in Figure 5 as an example to describe the entire route search and establishment process, as shown in Figure 12. For the convenience of showing the processing flow of the pipeline method, we assume that each processing unit takes the same amount of time and ignore the route establishment time. In practice, the time of each processing unit is different, but the processing flow of the pipeline is consistent.

**Figure 13:**
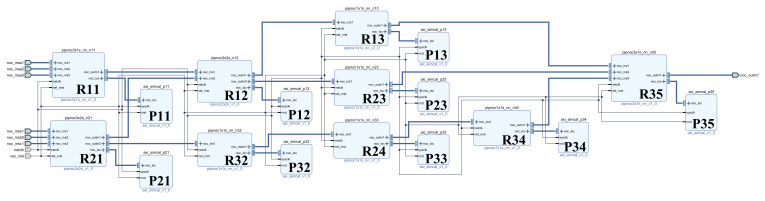
Structure diagram of the pipeline processing system.

**Figure 14:**
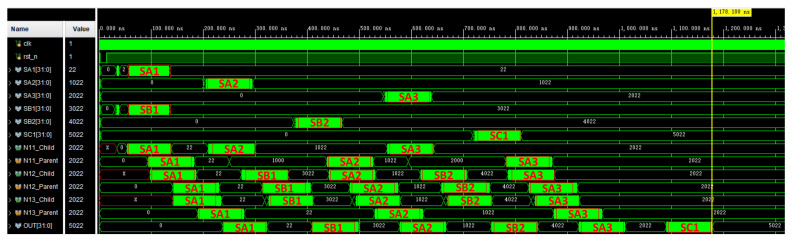
Simulation results of the pipeline processing system.

Based on the structure of Figure 5, we built a pipeline processing system as shown in Figure 13 in vivado to verify the pipeline processing function of the system. In order to facilitate verification, all processing units use multiplication and addition operation units with the same function and different IDs. Part of the simulation results are shown in Figure 14. The process of multi-signal processing is addressed, processed and transmitted according to the coordinate addressing method of our design of irregular topology. The figure shows the process of part of the signal passing through different signal processing units.

## 3. Experiment

In order to verify the effect in the actual scene, we designed a relatively common signal processing scene, as shown in Table 1. A total of 6 groups of signals with different sampling rates and characteristics underwent amplitude transformation, denoising reduction/low pass filters, and FFT (fast Fourier transform)/STFT (short-time Fourier transform).

Among them, SA1, SA2, SA3, SB1, and SB2 have the same sampling rate, and need to perform the same denoising reduction operation; SA1, SA2, SA3 have the same single processing data length, amplitude transformation operation (∗10), and the same FFT operation; SB1, SB2, SC1 have the same amplitude transform operation (∗20); and SB1, SB2 have the same single processing data length and the same STFT operation. The times below each processing operation are estimated processing times with a system clock of 250 MHz. We use the RSCPN, parallel structure and computer, respectively, to implement the above multi-signal processing, and then compare the resource consumption, execution time and execution results of the three methods.

We use the space-optimized resource allocation method to handle the above requirements:

$$\begin{array}{cc} T_{pkg} & =\{\frac{1024}{10\mathrm{Msps}},\frac{1024}{10\mathrm{Msps}},\frac{1024}{10\mathrm{Msps}},\frac{512}{10\mathrm{Msps}},\frac{512}{10\mathrm{Msps}},\frac{256}{1\mathrm{Msps}}\}\\ \; & =\{102.4\mathrm{us},102.4\mathrm{us},102.4\mathrm{us},51.2\mathrm{us},51.2\mathrm{us},256\mathrm{us}\}\\ t_{c} & =LCM(T_{pkg})=512\mathrm{us}\\ TPU_{p_{11}} & =4.418\mathrm{us}*\frac{512\mathrm{us}}{102.4\mathrm{us}}*3=66.27\mathrm{us}\\ TPU_{p_{12}} & =4.256\mathrm{us}*\frac{512\mathrm{us}}{102.4\mathrm{us}}*3+2.208\mathrm{us}*\frac{512\mathrm{us}}{51.2\mathrm{us}}*3=130.08\mathrm{us}\\ TPU_{p_{13}} & =12.818\mathrm{us}*\frac{512\mathrm{us}}{102.4\mathrm{us}}*3=192.27\mathrm{us}\\ TPU_{p_{21}} & =2.056\mathrm{us}*\frac{512\mathrm{us}}{51.2\mathrm{us}}*2+1.035\mathrm{us}*\frac{512\mathrm{us}}{256\mathrm{us}}=43.19\mathrm{us}\\ TPU_{p_{23}} & =6.584\mathrm{us}*\frac{512\mathrm{us}}{51.2\mathrm{us}}*2=131.68\mathrm{us}\\ TPU_{p_{32}} & =1.180\mathrm{us}*\frac{512\mathrm{us}}{256\mathrm{us}}*1=2.36\mathrm{us}\\ \forall TPUp_{i} & \leq t_{c}\times \theta =512\mathrm{us}*0.8=409.6\mathrm{us} \end{array}$$

**Figure 15:**
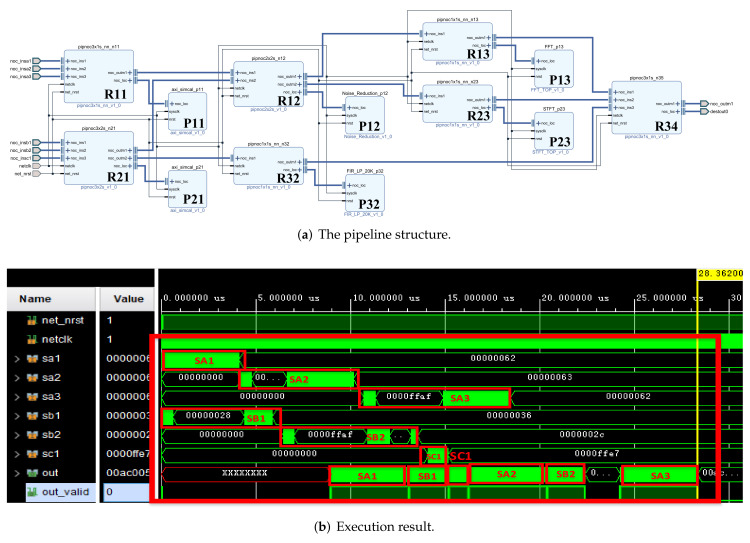
The pipeline structure and execution results for multi-signal processing in Table 1.

The allocation of all processing units met the requirements of the space-optimized resource allocation method. Then, we obtained the pipeline structure shown in Figure 15a, and the execution results are shown in the Figure 15b. The red text and boxes in the figure represent the input and output positions of the signal on the time axis.

**Figure 16:**
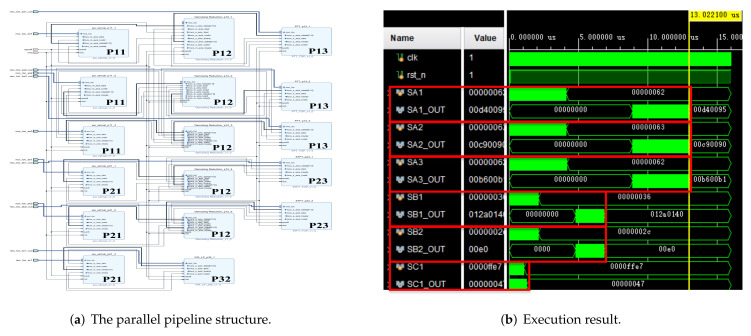
The parallel pipeline structure and execution results for multi-signal processing in Table 1.

In addition, we used the parallel pipeline method and the computer to realize the above-mentioned multi-signal processing. The structure diagram and simulation results of the parallel pipeline method are shown in Figure 16. The computer configuration is as follows: CPU I7-7700K, GPU GTX1060-6G, environment Matlab.

**Figure 17:**
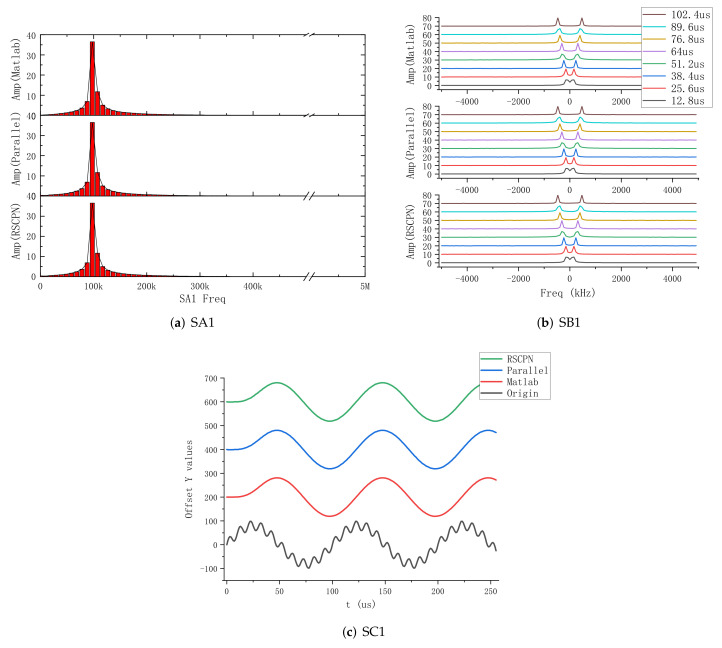
Partial signal results obtained by different processing methods.

For the convenience of comparison, we started from the beginning of a certain large period ($t_{c}$), at which time all signals were ready for a set of data to be processed, as shown in Figure 15b and Figure 16b At this time, the processing task was the heaviest, which better reflects the execution effect. Partial signal results were obtained by different processing methods, as shown in the Figure 17. The results obtained by the above 6 groups of signals through the three methods are consistent.

**Figure 18:**
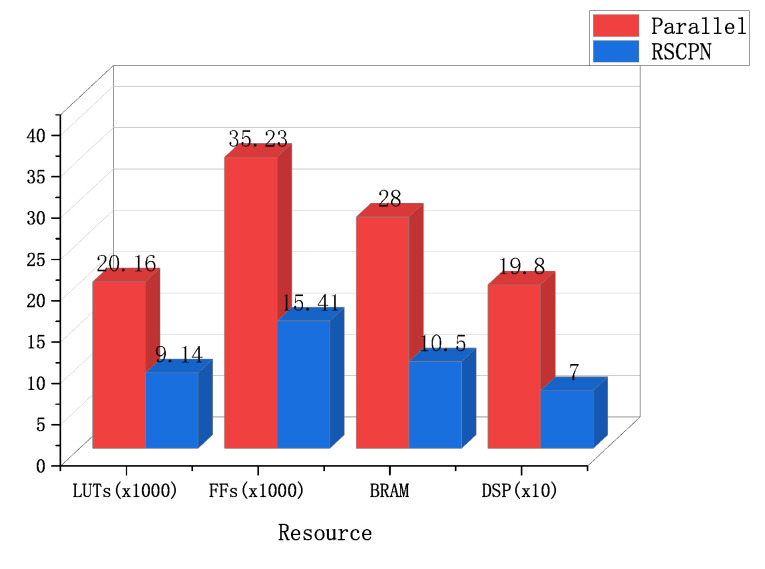
Comparison of resource occupancy of different methods.

**Figure 19:**
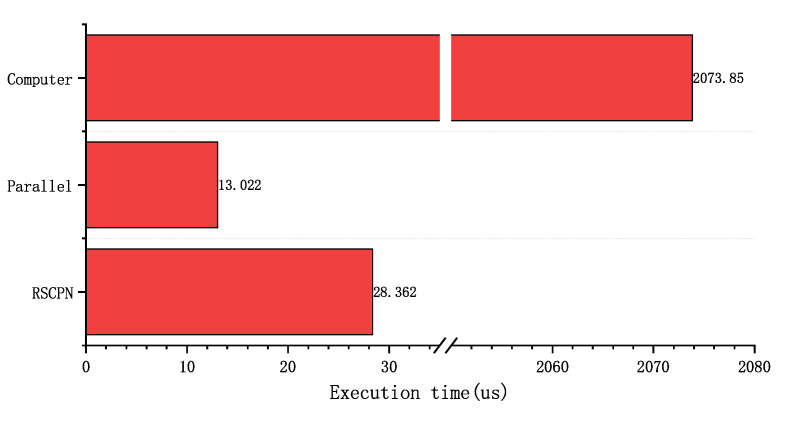
Execution time comparison of different methods.

From the point of view of resources, the method of using computer processing takes up the most resources. The resource comparison between RSCPN and the traditional parallel method is as shown in Figure 18. The resources occupied by RSCPN are far less than the traditional parallel methods. LUTs only occupy 45.3%, FFs only 43.7%, BRAM only 37.5%, and DSP only 35.4%. Under the premise of meeting the execution time requirements, the more the same processing units, the more obvious the effect of this resource saving will be. Figure 19 shows the execution time when different methods perform the busiest multi-signal processing operation. Before the arrival of the next valid data (appearance time of the next data packet of SB1 and SB2: 51.2 us), both RSCPN and traditional parallel processing methods have completed the above operations, and the computer did not complete the execution until 2073.85 us. Therefore, both RSCPN and traditional parallel processing methods can meet the requirements of real-time signal processing.

## 4. Disscusion

### 4.1. Compared with the Single-Chip Signal Processing System

Traditional single-chip signal processing systems collect and process data serially, while multi-channel signals are collected and processed by serial number polling. The RSCPN designed in this paper adopts the pipeline processing method, which dramatically improves data collection and processing ability and speed. The traditional single-chip signal processing system has a simple design suitable for situations with low sampling and a low number of signals. The RSCPN is more suitable for high-speed, multi-channel situations.

### 4.2. Compared with the Computer

The computer is more suitable for non-real-time large-scale signal processing, and its performance is relatively poor in real-time systems. Furthermore, the resources, cost, and power consumption of computers are not applicable to edge devices.

### 4.3. Compared with Traditional Parallel Processing System with FPGA

A traditional parallel processing system with FPGA is suitable for multi-channel, high-rate scenarios. However, as the number of signal processing channels increases, the resources occupied by this method are correspondingly doubled. The RSCPN designed in this paper can avoid this situation. However, with the increase in the number of signal processing channels, the resources occupied by the RSCPN designed in this paper will also increase, although the increase will be lower than that of the FPGA parallel signal processing system.

### 4.4. Compared with Network-on-Chip

The routing unit of the network-on-chip includes functions such as data buffering and more complex routing arbitration. It has a complex structure and occupies many resources. However, the pipeline routing unit structure in RSCPN does not cache data, and routing arbitration is relatively simple, occupying fewer resources.

### 4.5. Discussion of Flexibility

The RSCPN designed in this paper encapsulates commonly used signal processing operations in processing units and uses pipeline routing units to connect these signal processing operation units. Since the signal processing unit adopts the same design method, we can quickly carry out the secondary design through the configuration software according to the requirements. When the required changes are small, we can modify the input signal processing function sequence to complete the function change.

## 5. Conclusions and Extensions

With the rapid development of edge computing, the requirements for industrial condition monitoring, fault diagnosis, and health management are becoming more and more complex. Traditional serial and parallel signal processing architectures cannot resolve the conflict between increasingly complex processing requirements and resource-constrained edge devices. Therefore, we propose a resource-saving customizable pipeline network architecture for multi-signal processing in edge devices. This architecture reduces the resources required for multi-signal processing, further exploiting the performance potential of edge devices. This paper designs a space-optimized resource allocation method, which significantly reduces the resource requirements of multi-signal processing on the premise of meeting the real-time requirements. This paper designs a pipeline routing unit and a coordinate addressing method for irregular topology, which greatly reduces the resource consumption and time consumption in the routing process on the premise of ensuring flexibility and reliability. This architecture has positive significance for the performance improvement of edge devices and provides a new solution to the rapidly developing edge computing needs. In typical edge computing scenarios such as industrial site monitoring, fault diagnosis, and health management, this architecture can reduce the cost of edge devices and bring great economic benefits.

## Figures and Tables

**Table 1:** An instance of the need for multiple signal processing.

Signal ID	Sampling Rate	Amount of Data at One Time	Process 1	Process 2	Process 3
SA1	10 Msps	1024	P11:*10 (4.148 us)	P12:Denoising Reduction (4.256 us)	P13:FFT (12.818 us)
SA2	10 Msps	1024	P11:*10 (4.148 us)	P12:Denoising Reduction (4.256 us)	P13:FFT (12.818 us)
SA3	10 Msps	1024	P11:*10 (4.148 us)	P12:Denoising Reduction (4.256 us)	P13:FFT (12.818 us)
SB1	10 Msps	512	P21:*20 (2.056 us)	P12:Denoising Reduction (2.208 us)	P23:STFT (6.584 us)
SB2	10 Msps	512	P21:*20 (2.056 us)	P12:Denoising Reduction (2.208 us)	P23:STFT (6.584 us)
SC1	1 Msps	256	P21:*20 (1.035 us)	P32:Low Pass Filter (1.180 us)	

## Data Availability

Not applicable.

## References

[1] Abreha H.G., Hayajneh M., Serhani M.A. (2022). Federated Learning in Edge Computing: A Systematic Survey. Sensors.

[2] Liu D., Liang H., Zeng X., Zhang Q., Zhang Z., Minhong L. (2022). Edge Computing Application, Architecture, and Challenges in Ubiquitous Power Internet of Things. Front. Energy Res..

[3] Chen S., Eldar Y.C., Zhao L. (2021). Graph Unrolling Networks: Interpretable Neural Networks for Graph Signal Denoising. IEEE Trans. Signal Process..

[4] Yang F., Enzner G., Yang J. (2019). A Unified Approach to the Statistical Convergence Analysis of Frequency-Domain Adaptive Filters. IEEE Trans. Signal Process..

[5] Nadarajan S., Panda S.K., Bhangu B., Gupta A.K. (2016). Online Model-Based Condition Monitoring for Brushless Wound-Field Synchronous Generator to Detect and Diagnose Stator Windings Turn-to-Turn Shorts Using Extended Kalman Filter. IEEE Trans. Ind. Electron..

[6] Ruiz-Carcel C., Jaramillo V.H., Mba D., Ottewill J.R., Cao Y. (2015). Combination of process and vibration data for improved condition monitoring of industrial systems working under variable operating conditions. Mech. Syst. Signal Process..

[7] He J., Yang Q., Wang Z. (2020). On-line fault diagnosis and fault-tolerant operation of modular multilevel converters-A comprehensive review. CES Trans. Electr. Mach. Syst..

[8] Qin A., Hu Q., Lv Y., Zhang Q. (2019). Concurrent Fault Diagnosis Based on Bayesian Discriminating Analysis and Time Series Analysis With Dimensionless Parameters. IEEE Sens. J..

[9] Liu X., Song P., Yang C., Hao C., Peng W. (2017). Prognostics and Health Management of Bearings Based on Logarithmic Linear Recursive Least-Squares and Recursive Maximum Likelihood Estimation. IEEE Trans. Ind. Electron..

[10] Wang D., Tsui K.L., Miao Q. (2018). Prognostics and Health Management: A Review of Vibration Based Bearing and Gear Health Indicators. IEEE Access.

[11] Peng B., Wan S., Bi Y., Xue B., Zhang M. (2021). Automatic Feature Extraction and Construction Using Genetic Programming for Rotating Machinery Fault Diagnosis. IEEE Trans. Cybern..

[12] Jiang X., Zeng X., Sun J., Chen J. (2022). Distributed Solver for Discrete-Time Lyapunov Equations Over Dynamic Networks with Linear Convergence Rate. IEEE Trans. Cybern..

[13] Song W., Liu W., Pan Y. Design of Intelligent Rainwater Detection Window Based on STM32 Single-Chip Microcomputer. Proceedings of the 2019 Chinese Automation Congress (CAC).

[14] İlhan H.O., Aydin N. The contribution of DSP integration to ARM cores in SBCs for the video decoding process. Proceedings of the 2017 IEEE International Conference on Power, Control, Signals and Instrumentation Engineering (ICPCSI).

[15] Butt U.M., Khan S.A., Ullah A., Khaliq A., Reviriego P., Zahir A. (2021). Towards Low Latency and Resource-Efficient FPGA Implementations of the MUSIC Algorithm for Direction of Arrival Estimation. IEEE Trans. Circuits Syst. I Regul. Pap..

[16] Won J.Y., Lee J.S. (2018). Highly Integrated FPGA-Only Signal Digitization Method Using Single-Ended Memory Interface Input Receivers for Time-of-Flight PET Detectors. IEEE Trans. Biomed. Circuits Syst..

[17] Gul S., Siddiqui M.F., Rehman N.u. (2020). FPGA-Based Design for Online Computation of Multivariate Empirical Mode Decomposition. IEEE Trans. Circuits Syst. I Regul. Pap..

[18] Guner K.K., Gulum T.O., Erkmen B. (2021). FPGA-Based Wigner?Hough Transform System for Detection and Parameter Extraction of LPI Radar LFMCW Signals. IEEE Trans. Instrum. Meas..

[19] Weiyi S., Di Y., Xiaoyu L., Ke S. Parallelization method of digital signal processing based on multi-core pipeline. Proceedings of the 2017 IEEE 17th International Conference on Communication Technology (ICCT).

[20] Gou Y., Tang Y., Du X., Huang Z. Multi-channel wideband signal full bandwidth synchronous acquisition method. Proceedings of the 2021 IEEE 4th Advanced Information Management, Communicates, Electronic and Automation Control Conference (IMCEC).

[21] Adda M., Peratikou A. (2017). Routing and Fault Tolerance in Z-Fat Tree. IEEE Trans. Parallel Distrib. Syst..

[22] Wang L., Liu L., Han J., Wang X., Yin S., Wei S. (2020). Achieving Flexible Global Reconfiguration in NoCs Using Reconfigurable Rings. IEEE Trans. Parallel Distrib. Syst..

[23] Bhanu P.V., Govindan R., Kattamuri P., Soumya J., Cenkeramaddi L.R. (2021). Flexible Spare Core Placement in Torus Topology Based NoCs and Its Validation on an FPGA. IEEE Access.

[24] Prabhu Prasad B.M., Parane K., Talawar B. Hy-BTree: An efficient Tree based topology for FPGA based NoC implementation. Proceedings of the 2021 IEEE International Conference on Electronics, Computing and Communication Technologies (CONECCT).

